# Production of *CMAH* Knockout Preimplantation Embryos Derived From Immortalized Porcine Cells Via TALE Nucleases

**DOI:** 10.1038/mtna.2014.15

**Published:** 2014-05-27

**Authors:** JoonHo Moon, Choongil Lee, Su Jin Kim, Ji-Yei Choi, Byeong Chun Lee, Jin-Soo Kim, Goo Jang

**Affiliations:** 1 Laboratory of Theriogenology and Biotechnology, Department of Veterinary Clinical Science, College of Veterinary Medicine and the Research Institute of Veterinary Science, Seoul National University, Gwanak-gu, Seoul, South Korea; 2 Department of Chemistry, National Creative Initiatives Research Center for Genome Engineering, Seoul National University, Gwanak-gu, Seoul, South Korea; 3 Institute of Green Bio Science & Technology, Seoul National University, Pyeong Chang, Kangwon do, Korea; 4 Emergence Center for Food-Medicine Personalized Therapy System, Advanced Institutes of Convergence Technology, Seoul National University, Gyeonggi-do, Korea

**Keywords:** *CMAH* knockout, immortalization, somatic cell nuclear transfer, transcription activator-like effector nuclease

## Abstract

Although noncancerous immortalized cell lines have been developed by introducing genes into human and murine somatic cells, such cell lines have not been available in large domesticated animals like pigs. For immortalizing porcine cells, primary porcine fetal fibroblasts were isolated and cultured using the human telomerase reverse transcriptase (*hTERT*) gene. After selecting cells with neomycin for 2 weeks, outgrowing colonized cells were picked up and subcultured for expansion. Immortalized cells were cultured for more than 9 months without changing their doubling time (~24 hours) or their diameter (< 20 µm) while control cells became replicatively senescent during the same period. Even a single cell expanded to confluence in 100 mm dishes. Furthermore, to knockout the *CMAH* gene, designed plasmids encoding a transcription activator-like effector nuclease (TALENs) pairs were transfected into the immortalized cells. Each single colony was analyzed by the mutation-sensitive T7 endonuclease I assay, fluorescent PCR, and dideoxy sequencing to obtain three independent clonal populations of cells that contained biallelic modifications. One *CMAH* knockout clone was chosen and used for somatic cell nuclear transfer. Cloned embryos developed to the blastocyst stage. In conclusion, we demonstrated that immortalized porcine fibroblasts were successfully established using the human *hTERT* gene, and the TALENs enabled biallelic gene disruptions in these immortalized cells.

## Introduction

Pigs are considered to be good biomedical models for researches such as xenotransplantation because of their many physiological similarities with humans.^1,2,3,4^ Somatic cell nuclear transfer (SCNT) with genetically modified somatic cells has been used to generate pig models via transgenesis.^5^ Typical gene modifications are ectopic expression or knockout of target genes.^6,7^ While many cloned piglets have been produced using ectopic expression, only three kinds of knockout piglets (α-galactosidase, cystic fibrosis, and interleukin-2 receptor) using homologous recombination have been born.^2,8,9^ Developing knockout pig models has been hampered to date because fibroblasts generally have a limited life span during *in vitro* culture and because of the low efficiency of the homologous recombination process.^4^ To overcome these two issues, immortalization of fibroblasts and more efficient knockout protocols are needed. For immortalization, several genes such as *BMI*, *SV40LT*, and human telomerase reverse transcriptase (*hTERT*) can be transfected into cells. In previous studies, *SV40LT* and *hTERT* were used to immortalize porcine cells.^10,11,12,13^

Transcription activator-like effector nuclease (TALEN) is an emerging high-end technology used to create targeted double-stranded breaks in DNA.^14^ Currently, TALEN has been employed for genome editing, resulting in target gene deletion or insertion in human, mouse, and rat cells.^15,16,17,18,19^ Application of TALEN to cells of large animals like pigs could more efficiently generate knockout cell lines and thus help to elucidate the underlying molecular processes.^20^ After establishing knockout cell lines, the cell nuclei could potentially be reprogrammed in enucleated oocytes and produce knockout cloned offspring.

In this study, to prove TALEN-mediated knockout, we elected to delete the *CMAH* gene, which is another important cell surface glycoprotein with α-galactosidase, for xenotransplantation pig models, and then, gene knockout cells were used for feasibility of embryonic development via SCNT. Here, we hypothesized that using immortalization and TALEN approaches together in porcine cells could serve as practical *in vitro* models of genome editing.

## Results

### Cell properties

**Figure 1:**
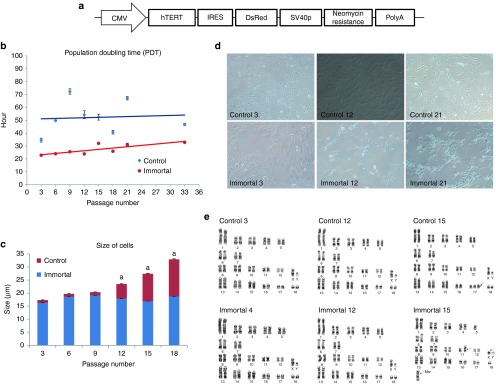
**Cellular analysis of porcine immortalized cells. **(**a**) Illustration of *pCMV-hTERT-IRES-DsRed*, (**b**) Population doubling time, significant differences in doubling time between control and immortalized cell were investigated, and those were 46.4 ± 1.1 and 26.9 ± 0.6, respectively. (**c**) Size differences between control cells and immortalized cells, mean cell size of the immortalized cells were 17.9 ± 0.2 which was constantly under the 20 µm while that of control cells were sequentially increasing in mean cell size until these cells enter into senescence or crisis. Significant differences in cell size between control and immortalized cells were observed from passage number 12. (**d**) Morphologies of control cells and immortalized cells, numbers represent passages. (**e**) Results of karyotyping, both control and immortalized cells, showed abnormalities from passage number 15. Arrows indicate the abnormal site in chromosomes.

*Differences between controls and immortalized cells in morphology, doubling times, and cell size.* After transfection, outgrowing colonized fibroblasts were cultured. Along with immortalization of the cells, their size was reduced (**Figure 1d**).

Mean doubling time of control and immortalized cells were 46.4 ± 1.1 and 26.9 ± 0.6 hours, respectively, and these values were significantly different (*P* < 0.05; **Figure 1b**). Mean cell size of the immortalized cells was 17.9 ± 0.2 µm and always less than 20 µm, while mean cell size of control cells was progressively increasing until these cells enter into replicative senescence. Significant differences in cell size between control and immortalized cells were observed from passage 12 (**Figure 1c**).

**Figure 2:**
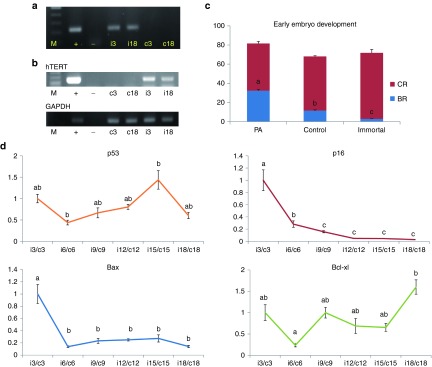
**Gene expression in immortalized cells and embryonic development.** (**a**) Detection of *hTERT* (M, marker; +, positive control vector; −, negative control vector; c, control cells; i, immortalized cells; and numbers referred to passages). (**b**) Expression of *hTERT* (M, marker; +, positive control vector; −, negative control vector; c, control cells; i, immortalized cells; and numbers referred to passages). (**c**) Early embryonic development: changes among early embryonic development when cell properties were changed into immortal states. PA referred parthenogenetic activation. Those control and immortal indicated SCNT results when the donor cell were control and immortalized cells, respectively. Cleavage rates (CRs) were not changed among groups, but blastocyst formation (BR) rates were serially significantly decreased among three groups. (**d**) Gene expression: tumor suppressor gene (*p53*) expression level was not significantly changed during the increasing of passage number in immortalized cells/control cells. Cyclin-dependent kinase inhibitor 2A (*p16*) expression were significantly downregulated during the increasing of passage numbers in immortalized cells/control cells. Also *Bax*, which is well known for proapoptotic gene, were significantly downregulated during the increasing of passage numbers in immortalized cells/control cells. However, *Bcl-xl*, antiapoptotic gene, was significantly upregulated during the increasing of passage numbers in immortalized cells/control cells. *hTERT*, human telomerase reverse transcriptase; SCNT, somatic cell nuclear transfer.

*PCR, RT-PCR, and sequencing.* Integration and expression of the *hTERT* gene was observed by genomic DNA PCR and RT-PCR in 3 and 18 passages of control and immortalized cells, respectively. PCR and RT-PCR data indicated that the *hTERT* gene was integrated into immortalized cells (**Figure 2a**,**b**). Also, sequencing results from both PCR amplificons were exactly the same as those inserted sequences from the vector (see **Supplementary Figure S3**).

*Karyotyping.* A total of 20 cells in each analysis were subjected on karyotyping. Karyotyping of immortalized cells, prior to passage number 15, revealed normal chromosomes, while subsequently abnormal chromosomes were detected in one cell (**Figure 1e**). Similar observations were made in control cells, indicating that eight cells showed abnormalities (trisomy in chromosome #17) (**Figure 1e**).

### Single cell colony formation

Immortalized cells could be populated from a single cell in a 100 mm dish. This ability was replicated three times more using single cells. However, control cells did not have the ability to be expanded from a single cell in a 100 mm dish (see **Supplementary Figure S1**).

### Gene expression

Gene expression in immortalized cells and control cells are summarized in **Figure 2d**. In this analysis, tumor suppressor gene (*p53*) expression level was not significantly changed during increasing passage number in immortalized or control cells except passage numbers 6 and 15. Cyclin-dependent kinase inhibitor 2A (*p16*) expression was significantly downregulated during increasing passages (from passage 6) in immortalized cells. Also, *Bax*, which is a well-known proapoptotic gene, was significantly downregulated during increasing passage numbers (from passage 6) in immortalized cells. However, *Bcl-xl*, an antiapoptotic gene, was significantly changed in passage numbers 6 and 18. In the analysis of metabolic genes, expression of glucose transporter 1 (*GLUT1*) and lactate dehydrogenase A (*LDHA*) were significantly upregulated in late passage of immortalized and control cells, respectively. Expression of methylation relation genes (DNA methyltransferase (*DNMT*)*1*, *DNMT3a*, and *DNMT3b*) was not changed (see **Supplementary Figure S2**).

### Telomerase activity test

Relative telomerase activity (RTA) of control and immortalized cells were 0.41 ± 0.16 and 5.37 ± 0.09, respectively. Telomerase activity was significantly increased in immortalized cells compared with control cells (**Figure 4**).

### Preimplantation development of cloned embryos derived from immortalized cells

Development rates were evaluated in three groups: parthenogenetically activated embryos (total numbers of oocytes: 211), SCNT-derived embryos using control somatic cells as nuclear donors (total numbers of oocytes: 112), and SCNT-derived embryos using immortalized cells as nuclear donors (total numbers of oocytes: 107). Three replicates were done in all three groups. Two days after activation, cleavage rates evaluated under a microscope were 81.6 ± 2.2, 68.1 ± 0.8, and 71.8 ± 3.6%, respectively. No significant differences were observed in cleavage rate among the three groups. However, significant differences were observed in blastocyst formation rates, which were 32.3 ± 1.2, 11.5 ± 0.7, and 2.9 ± 0.2%, respectively (**Figure 2c**).

### *CMAH* knockout and SCNT

**Figure 3:**
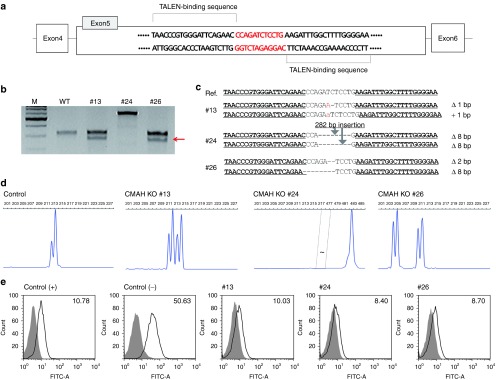
**Generating CMAH knockout cells and its analysis. **(**a**) DNA-binding sequences and the spacer region for *CMAH*-TALEN. (**b**) T7 endonuclease I (T7E1) assays: T7E1 assays were conducted using genomic DNA from three *CMAH* knockout clones. The arrow indicates the size (~170 bp) of T7E1-digested DNA fragments. (**c**) DNA sequences of the *CMAH* locus from each *CMAH* knockout clone. “−” denotes deleted nucleotides. Red colored upper case letter and lower case letter sequences represent nucleotide substitutions and insertion, respectively. (**d**) Fluorescent PCR (fPCR) assay of the *CMAH* knockout clones. (**e**) FACS analysis of *CMAH* knockout clones. The expression level of *N*-glycolylneuraminic acid (Neu5Gc) is detected by anti-Neu5Gc antibody on the *CMAH* knockout cell membrane. The expression levels of Neu5Gc on each *CMAH* knockout clone are comparable with control (+). Control (+), human embryonic kidney cell line; control (−), nontransfected porcine fibroblasts; FACS, fluorescence-activated cell sorting; TALEN, transcription activator-like effector nuclease.

After transfecting TALEN DNAs, 500 reporter gene–positive cells were cultured in a 100 mm dish and grown into colonies; 116 single cell–derived colonies were selected. In a T7E1 mutation assay, we found 45 colonies to be mutated (see **Supplementary Figure S4**). These 45 colonies were subjected to fluorescent PCR for determination of biallelic mutated colonies (see **Supplementary Figure S5**). Three biallelic mutation colonies with morphologically good cells were finally selected (**Figure 3d**) and sequenced for confirmation of biallelic mutation. In #13, a 1 bp insertion and 1 bp deletion were found; in # 24, a 282 bp insertion (sequence of 282 bp was noted in **Supplementary Figure S6**) and in #26, 2 and 8 bp deletions were observed (**Figure 3c**). In addition, as shown by fluorescence-activated cell sorting, *CMAH* expression was removed in all three cell lines (**Figure 3e**). Furthermore, 36 cloned embryos derived from *CMAH* knockout cells were reprogrammed after insertion into enucleated oocytes, cleaved (91.7%), and developed into a blastocyst (2.8%) (see **Supplementary Figure S7**).

## Discussion

It is well established in humans and in mice that cell lines are necessary to understand or evaluate the molecular process.^21,22,23^ However, in pigs, such research has been limited to date. In this study, we developed immortalized cells, and furthermore, those cells were used for TALEN to knockout the interesting genes.

For inducing immortalization, genes such as *SV40LT*, *BMI*, and *hTERT* were used in previous studies.^24^ Among these genes, *hTERT* has especially been used to immortalize cells because of reduced chromosome damage.^24,25^ In this study, *hTERT* successfully induced pig fetal fibroblasts into immortalization. In addition, our immortalized cells can be cultured for single cell colony formation at least three times. Therefore, single immortalized cell was cultured and propagated to unlimited numbers, indicating that single mutated cells can be isolated and used for many assays requiring cells, DNAs, RNAs, and proteins.

**Figure 4:**
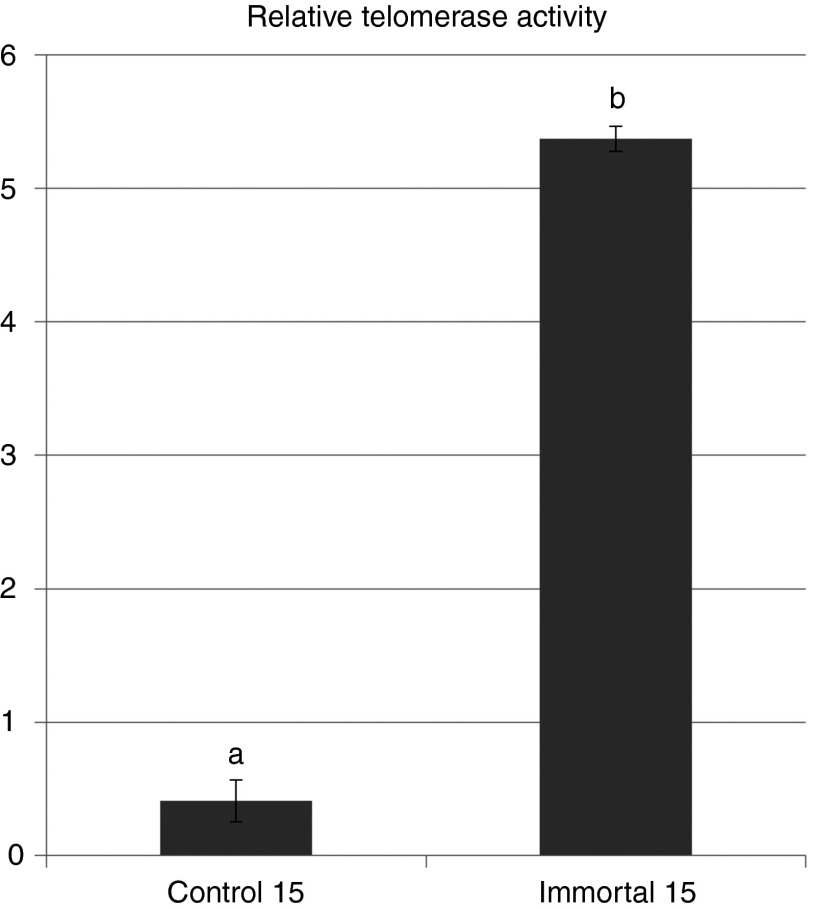
Relative telomerase activity (RTA). RTA of control and immortalized cells were 0.41 ± 0.16 and 5.37 ± 0.09, respectively. Telomerase activity was significantly increased in immortalized cells compared with control cells.

For investigating properties of the immortalized cells, expression of proliferative, apoptotic, metabolic, and methylation-related genes were analyzed. Even though significant change of *p53* expression at specific passage was observed during long-term culture, *p16* was dramatically downregulated (**Figure 2**) from passage 6. The fact that loss of *p16* function after transfection with *hTERT* is much related to immortalization is in line with our results.^26^ As expected, antiapoptotic gene (*Bax*) was increased but proapoptotic gene (*Bcl-xl*) was decreased. In metabolic gene expression, *GLUT1* was significantly increased because immortalized cell utilize more glucose for unlimited cell proliferative competence like cancer cells.^27^ Moreover, expression of *LDHA*, which is soluble cytosolic enzyme resulting from apoptosis or necrosis, was increased in late-passage control cells.^28^ One point is about methylation gene expression. In contrast to our results, a previous study reported increase of *DNMT1* expression in human fibroblast after *hTERT* transfection.^29^ However, in our case, the *DNMTs* expression levels were not changed after immortalization. In this study, we found that downregulation (*p16* and *Bax*) and upregulation (*GLUT1* and telomerase activity) plays an important role in maintaining the unlimited cell propagation (**Figures 2d** and **4**; see **Supplementary Figure S2**).

Additionally, it has raised a scientific interest on SCNT embryo production using immortalized cells with long-term culture properties. With respect to early embryonic development, no significant differences were observed in cleavage rates among PA embryos, SCNT embryos derived with control cells, and SCNT embryos derived with immortalized cells. However, significant differences were observed in blastocyst formation rates among these three groups. In particular, blastocyst formation rates were significantly decreased in the immortalized cell SCNT group. The very low embryonic development using immortalized cells is similar to that reported in a previous study, in which immortalized bovine epithelial cells used in SCNT could not support embryo development into blastocysts.^30^ However, with ovine immortalized fibroblasts using *hTERT* as in this study, there was no significant difference between control and immortalized groups in their ability to support SCNT early embryo development.^31^ In two previous studies,^30,31^ it was concluded that transformed cells with abnormal cellular responses (like serum starvation) failed to support embryonic development into blastocysts. Therefore, we assume that morphological and proliferative changes in our cells immortalized using *hTERT* affected normal cellular gene expression profiles including p16 and resulted in very low embryonic development. Because of short cell doubling time, it is hard to get exact G0/G1 cells from immortalized cells. This is another possible reason why SCNT embryos using immortalized cells could not well developed to blastocysts.

TALEN, an emerging genome editing tool, can be applied to generate mutant pigs. To knockout a gene using TALEN, several pairs on a specific coding domain region should be designed and evaluated for choosing the most effective pairs. Thus, effective TALEN DNA pairs deleted the DNA with an efficiency of 3.9–43% to date.^14^ However, validation systems to determine effective pairs in different species could provide different genome editing efficiencies.^32^ In fibroblasts, before homogeneous knockout cell lines were achieved, single isolated mutated cells became senescent and thus could not be subjected to further analysis and application. Therefore, we strongly suggest that in porcine genome editing, these immortalized cells could be used as appropriate *in vitro* test cell lines to select effective pairs of TALEN.

Because immortalized cell line can be grown up from a single cell to billions of cells, we randomly chose 100 single cells and cultured them into colonies of homogeneous s cells. Using single cell colony formation competence as a selection criterion, we can more easily generate knockout cell lines. Indeed, in this study, single cell colonies grew well, and these were used to analyze each colony to evaluate mutation characteristics. As a result, TALEN activity showed 38.8% efficiency, and many cell lines were isolated (see **Supplementary Figure S4**). From the finally chosen cells, three biallelic mutated cell lines developed that did not express the cell surface carbohydrate chain, *N*-glycolylneuraminic acid (Neu5Gc). From one of these three mutated cell lines, colony number 24, cells were used as nuclear donor cells for SCNT. Although the blastocyst formation rate was low, we observed that immortalized cells with *CMAH* knockout can be reprogrammed in porcine enucleated oocytes and develop to the blastocyst stage. Additionally, the other gene, *GGTA1*, which is responsible for hyperacute rejection in xenotransplantation, was also knocked out in this cell line using exactly the same methods with high efficiency (see **Supplementary Figures S8–S12**).^4^

In conclusion, the *hTERT* gene prolonged the usual life span of porcine fibroblasts into immortalized status. Immortalized cells with single cell survival properties were treated with TALEN to delete *CMAH*. Then, knocked out cells were employed to generate preimplantation embryos. These immortalized cells must become useful tools as an *in vitro* model to select the most effective TALEN pairs and knockout-specific genes to support development of biomedically useful pig models.

## Materials and methods

All chemicals were obtained from Sigma-Aldrich (St. Louis, MO) unless otherwise stated.

*Primary cell culture and maintenance.* Male fetal fibroblasts from one miniature pig fetus, which were used as control cells, were isolated and cultured. Euthanized fetus was dissected into three parts: head, body, and tail. Just the body parts of fetuses were washed three times in phosphate-buffered saline and then chopped into small pieces in a 60 mm dish with trypsin. Trypsinized tissues were then incubated for 30 minutes at 37 °C. Well-dissociated tissues were centrifuged at 1,500 rpm for 2 minutes. The supernatant was discarded, and the pellet was resuspended with phosphate-buffered saline and then centrifuged at 1,500 rpm for 2 minutes. These procedures were repeated two times. Finally, the supernatant was discarded, and the pellet was resuspended in Dulbecco's Modified Eagle's Medium (DMEM; Gibco, Carlsbad, CA) supplemented with 15% fetal bovine serum (Gibco), 1% Penicillin/Streptomycin (P/S; Gibco), 1% nonessential amino acid (NEAA; Gibco), and 100 mmol/l β-mercaptoethanol (β-ME) by inverting the tube several times. The cells resuspended in this medium were held at room temperature (~25 °C) for 5 minutes, and then, the suspension was transferred into a cell culture dish for ~10 days with culture medium changed every 2–3 days. These primary cells were cultured, expanded, and frozen at −196 °C for further use. The cell cultures were maintained in DMEM with 15% fetal bovine serum, 1% P/S, 1% NEAA, and 100 mmol/l β-ME.

*Immortalization.* For immortalization, *hTERT* (from Addgene, http://www.addgene.org/, Plasmid #12245) were amplified by PCR. Purified *hTERT* fragments were inserted in *pCMV-IRES-DsRed* vectors, which were purchased from Clontech (Seoul, Korea.). *pCMV-hTERT-IRES-DsRed* plasmids were transfected into male fetal fibroblasts which was same cells as control cells using FugeneHD (**Figure 1a**). Two days after transfection, 1,000 μg/ml neomycin (G418; Gibco) were treated for 7 days to isolate the transfected cells and then growing cells to neomycin resistance were subcultured (**Figure 1**).

### Cell properties

*Doubling time.* Controls and immortalized cells were plated in 12-well plates at 4 × 10^4^ cells/well. Every 24 hours, cells in four of the wells were trypsinized, and cell numbers were calculated manually under a hemocytometer. Then, the doubling time was calculated using the doubling time online calculator (http://www.doubling-time.com/compute.php)^33^ every 3 passages up to passage 21, and passage 33 was evaluated as well.

*Cell size.* Images from trypsinized cells on the hemocytometer were taken under a microscope (×200). Sizes of 100 cells were measured by ImageJ (http://rsbweb.nih.gov/ij/)^34^ every 3 passages up to passage 21, and passage 33 was evaluated as well.

*PCR.* Genomic DNA was extracted with the G-spin Genomic DNA Extraction Kit (iNtRON Biotechnology, Gyeonggi-do, Korea) according to the manufacturer's protocol. Amplification of target genes was achieved using Maxime PCR PreMix (i-StarTaq, iNtRON). Primer sets, conditions, and expected sizes are annotated in **Supplementary Table S1**.

*Sequencing.* Target DNA samples were delivered to a sequencing company (Macrogen, Seoul, Korea). Briefly, sequencing reactions were performed in the DNA Engine Tetrad 2 Peltier Thermal Cycler (BIO-RAD, Seoul, Korea) using the ABI BigDye (R) Terminator v3.1 Cycle Sequencing Kit (Applied Biosystems, Seoul, Korea), following the protocols supplied by the manufacturer. Single-pass sequencing was performed on each template using a selected primer (primer sequences: AACGTTCCGCAGAGAAAAGA). The fluorescent-labeled fragments were purified by the method recommended by Applied Biosystems because it removes unincorporated terminators and dNTPs (dNTP indicates the mixture of dATP, dCTP, dGTP and dTTP). The samples were subjected to electrophoresis in an ABI 3730xl DNA Analyzer (Applied Biosystems).

*Karyotyping.* To perform karyotyping, cultured cells were treated as follows. First, 200 μl of colcemid (Gibco) stock solution was added to the culture. Then, the culture was returned to the incubator (37 °C, 5% CO_2_) for 4 hours. After incubation, cells were collected in 15 ml tubes and then centrifuged at 1,000 rpm for 10 minutes. The medium was carefully aspirated, and then, 5 ml of hypotonic solution (0.075 mol/l KCl) was added and allowed to stand at 37 °C for 10 minutes. Then, 500 μl of Carnoy's fixative (methanol:acetic acid 3:1) was added and mixed by inverting the tube, followed by centrifugation at 1,000 rpm for 10 minutes. The hypotonic solution was aspirated carefully, and 3 ml of Carnoy's fixative was added and mixed well. After more than 20 minutes, the mixture was centrifuged at 1,000 rpm for 10 minutes. The supernatant fixative solution was carefully aspirated till leaving about two times of volume to pellets. The pellet was spread on a prepared glass slide which was then baked at 60 °C for 30 minutes. The slide was treated with 50% H_2_O_2_ for 3 minutes, then baked again at 60 °C for 30 minutes. Finally, the slide was stained with the Giemsa stain-GTG banding method. Chromosome imaging were accomplished with the ChIPS-Karyo (Chromosome Image Processing System; GenDix, Seoul, Korea).

*Single cell colony formation.* Trypsinized cells were placed on the lid of Falcon dish (Catalog number #351006; Falcon, Franklin Lakes, NJ) in drops of 20 μl of DMEM containing 15% fetal bovine serum, 1% P/S, 1% NEAA, and 100 mmol/l β-ME. To evaluate single cell colony-forming competence, one cell was picked up in a micropipette attached to a micromanipulator. The cell was transferred into a 4 μl drop of DMEM containing 15% fetal bovine serum, 1% P/S, 1% NEAA, and 100 mmol/l β-ME that was covered with mineral oil. After 7 days, growing cell colonies were collected and sequentially subcultured into 96-, 24- and 6-well plates. Then, cells from the 6-well plates were moved sequentially to 60 and 100 mm dishes.

*Gene expression.* Total RNAs were extracted to analyze gene expression in the immortalized cells by using the easy-spin Total RNA Extraction Kit (iNtRON). Then, complementary DNAs (cDNAs) were synthesized using Maxime RT Premix (iNtRON) according to the manufacturer's protocol. Information on primers is listed in **Supplementary Table S2**. Gene expression for *p53, p16, Bax, Bcl-xl, DNMT1, DNMT3a, DNMT3b, GLUT1*, and *LDHA* was measured with a RT-PCR machine (7300 Real-Time PCR System; Applied Biosystems).

*Telomerase activity test.* Quantification and characterization of telomerase activity was done by the telomeric repeat amplification protocol. For this test, TeloTAGGG Telomerase PCR ELISA^PLUS^ (Roche, Basel, Switzerland) kit was used with manufacturer's indications.^35^ RTA within different samples in an experiment were obtained using the following formula: RTA = {(AS-AS0)/AS,IS}/{(ATS8-ATS8,0)/ATS3,IS} × 100 (AS; absorbance of sample, AS,0; absorbance of heat-treated sample, AS,IS; absorbance of internal standard (IS) of the sample, ATS8; absorbance of control template, ATS8,0; absorbance of lysis buffer, ATS8,IS; absorbance of IS of the control template).

*Nuclear transfer.* Donor cells were subjected to nuclear transfer, which was done following the protocol previously established in our studies.^36^ Briefly, immature oocytes were obtained from pig ovaries and cultured for 40 hours to support maturation. The *in vitro* matured oocytes were enucleated using an aspiration pipette, then microinjected with a control or transfected donor cell, fused by electrical stimulation, and activated using an electrical protocol. The resulting activated embryos were cultured for 7 days. Cleavage and blastocyst stages were observed on days 2 and 7 of culture, respectively.

*CMAH knockout using TALEN and magnetic separation.* All TALEN plasmids were obtained from ToolGen (ToolGen, Seoul, Korea).^37^ 1 x 10^6^ immortalized cells were transfected using 30 μl of Turbofect (Fermentas, Glen Burnie, MD) and 10 μg of plasmid DNA at a weight ratio of 45:45:10 (plasmid encoding a TALEN:plasmid encoding the other TALEN:magnetic reporter) according to the manufacturer's protocol.^15,38^ The transfected cells were cultured for 2 days at 37 °C and subjected to magnetic separation. Trypsinized cell suspensions were mixed with magnetic bead-conjugated antibody against H-2K^k^ (MACSelect K^k^ microbeads; Miltenyi Biotech, Cologne, Germany) and incubated for 15 minutes at 4 °C. Labeled cells were separated using a column (MACS LS column; Miltenyi Biotech, Germany) according to the manufacturer's protocol.

*T7E1 assay.* Genomic DNA was extracted using the G-DEX IIc Genomic DNA Extraction Kit (iNtRON) after 3 days of transfection. TALEN target sites were PCR amplified using primer pairs listed in **Supplementary Table S1**. The T7E1 analysis was done as described previously.^38,39^ The amplicons were denatured by heating and annealed to form heteroduplex DNA, which was treated with 5 units of T7 endonuclease 1 (New England Biolabs, Ipswich, MA) for 20 minutes at 37 °C and then analyzed by 2.5% agarose gel electrophoresis.

*Fluorescent PCR.* Carboxyfluorescein was labeled on 5′ end of the forward primer by an oligo synthesis company (Bioneer, Daejon, South Korea). PCR products were processed for fragment separation by capillary electrophoresis on an ABI 3730xl using POP-7 polymer. The GeneScan Rox500 size standard (Life Technologies, Grand Island, NY) was run as an internal size marker. Samples were denatured at 95 °C for 5 minutes and run on the genetic analyzer. Data were analyzed for allele sizes and peak heights using the pick scanner software v1.0 (Life Technologies).

*Fluorescence-activated cell sorting. CMAH* biallelic knockout cells were trypsinized and resuspended in staining buffer (0.1% bovine serum albumin in phosphate-buffered saline) to reach a final concentration of 5 × 10^5^ to 1 × 10^6^ cells/ml. The cells were incubated for 20 minutes on ice with the antibody anti-Neu5Gc (Sialix, Waban, MA). After incubation, the cells were washed twice with staining buffer and resuspended, then the stained cells were analyzed by fluorescence-activated cell sorting.

*Statistical analysis.* All data were analyzed by one-way ANOVA followed by Tukey's multiple comparison test or paired *t*-test using GraphPad Prism version 5.01 (http://www.graphpad.com/scientific-software/prism/) to determine differences among experimental groups. Statistical significance was determined when the *P* value was less than 0.05.

**SUPPLEMENTARY MATERIAL** **Table S1.** List of primers. **Table S2.** List of real-time PCR primers. **Figure S1.** Single cell culture of immortalized cell. **Figure S2.** Gene expression analysis **Figure S3.** Sequencing results from genomic DNA from immortalized cell lines. **Figure S4.** T7E1 assay results from *CMAH* KO single cell colonies. **Figure S5.** Fluorescent PCR results from *CMAH* KO single cell colonies. **Figure S6.** Inserted 282 bp sequences in #24 colony. **Figure S7.** SCNT with *CMAH* KO donor cells. **Figure S8.** Illustration of TALEN binding sitesand results of *GGTA1*-TALEN KO. **Figure S9.** T7E1 assay results from 1^st^ *GGTA1* KO single cell colonies. **Figure S10.** Fluorescent PCR results from 1^st^ *GGTA1* KO single cell colonies. **Figure S11.** T7E1 assay results from 2^nd^ *GGTA1* KO single cell colonies. **Figure S12.** Fluorescent PCR results from 2^nd^ *GGTA1* KO single cell colonies.

## Supplementary Material

Supplementary Figure S1 Single cell culture of immortalized cell.

Supplementary Figure S2 Gene expression analysis

Supplementary Figure S3 Sequencing results from genomic DNA from immortalized cell lines.

Supplementary Figure S4 T7E1 assay results from *CMAH* KO single cell colonies.

Supplementary Figure S5 Fluorescent PCR results from *CMAH* KO single cell colonies.

Supplementary Figure S6 Inserted 282 bp sequences in #24 colony.

Supplementary Figure S7 SCNT with *CMAH* KO donor cells.

Supplementary Figures S8 Illustration of TALEN binding sitesand results of *GGTA1*-TALEN KO.

Supplementary Figures S9 T7E1 assay results from 1^st^ *GGTA1* KO single cell colonies.

Supplementary Figures S10 Fluorescent PCR results from 1^st^ *GGTA1* KO single cell colonies.

Supplementary Figures S11 T7E1 assay results from 2^nd^ *GGTA1* KO single cell colonies.

Supplementary Figures S12 Fluorescent PCR results from 2^nd^ *GGTA1* KO single cell colonies.

Supplementary Table S1 List of primers.

Supplementary Table S2 List of real-time PCR primers.
