# Supplementary material for: Production of CMAH Knockout Preimplantation Embryos Derived From Immortalized Porcine Cells Via TALE Nucleases
Source: Mol Ther Nucleic Acids. 2014 May 27;3(5):e166–. doi: 10.1038/mtna.2014.15 (PMC4040627; doi:10.1038/mtna.2014.15)
Supplement: Supplementary Figure S1 — Single cell culture of immortalized cell. [file mtna201415x1.doc]

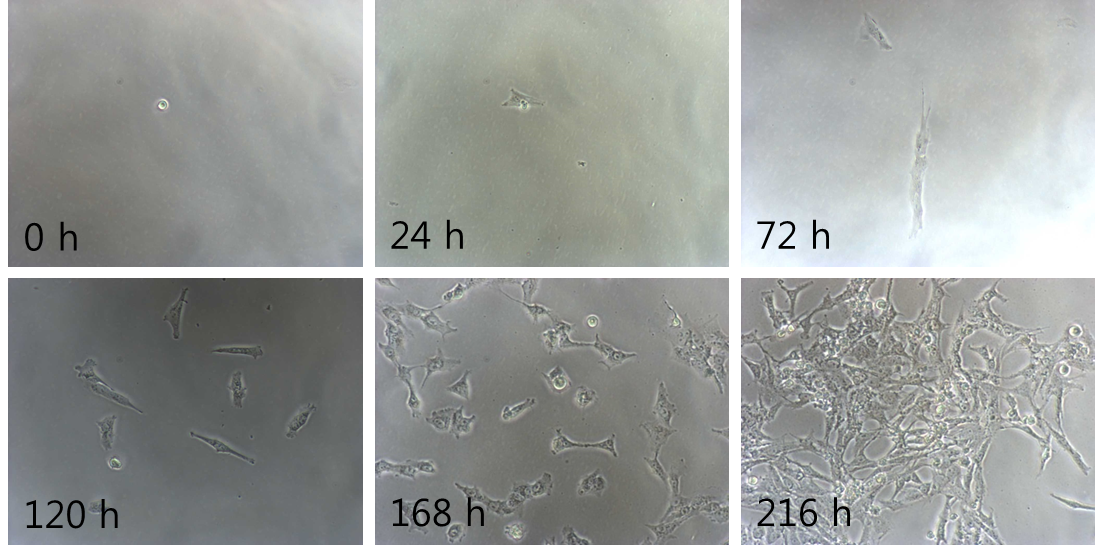


**Figure S1. Single cell culture of immortalized cell.**

Single cell in 4 μl drop of culture media were observed by under-left listed time. One colony was established from one cell.
