# Supplementary material for: Production of CMAH Knockout Preimplantation Embryos Derived From Immortalized Porcine Cells Via TALE Nucleases
Source: Mol Ther Nucleic Acids. 2014 May 27;3(5):e166–. doi: 10.1038/mtna.2014.15 (PMC4040627; doi:10.1038/mtna.2014.15)
Supplement: Supplementary Figure S2 — Gene expression analysis [file mtna201415x2.doc]

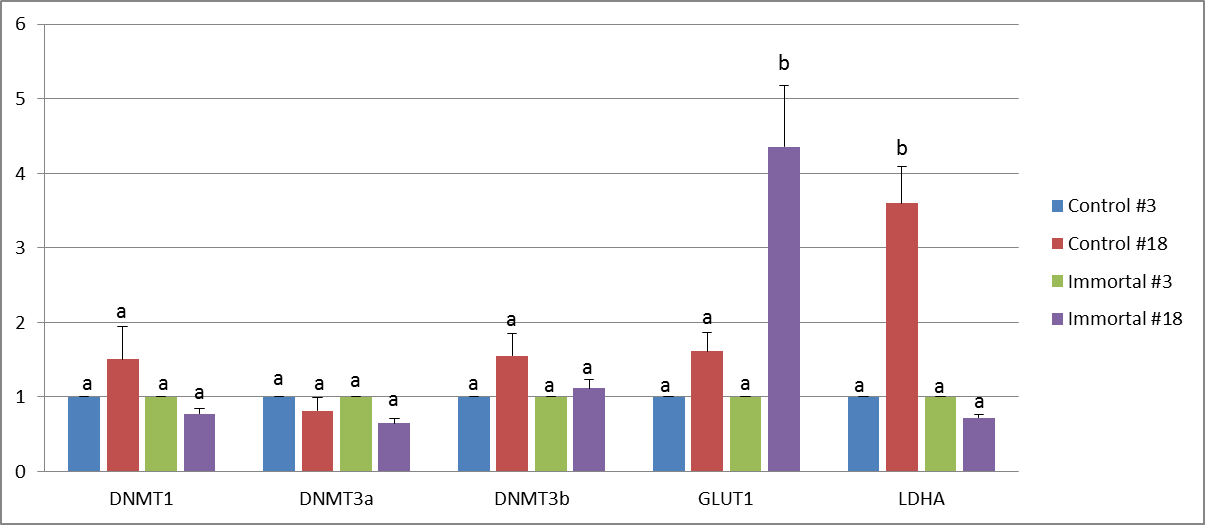


**Figure S2. Gene expression analysis.** Methylation related DNA methyltransferase (DNMT)1, DNMT3a, and DNMT3b genes were evaluated. No significant differences were observed in methylation patterns of control cell lines between immortalized cell lines at any passages. However, glucose transporter 1 (GLUT1) and lactate dehydrogenase A (LDHA) those genes were related to metabolism were also evaluated.
