# Supplementary material for: Production of CMAH Knockout Preimplantation Embryos Derived From Immortalized Porcine Cells Via TALE Nucleases
Source: Mol Ther Nucleic Acids. 2014 May 27;3(5):e166–. doi: 10.1038/mtna.2014.15 (PMC4040627; doi:10.1038/mtna.2014.15)
Supplement: Supplementary Figure S3 — Sequencing results from genomic DNA from immortalized cell lines. [file mtna201415x3.doc]

**Figure S3. Sequencing results from genomic DNA from immortalized cell lines.**
