# Supplementary material for: Production of CMAH Knockout Preimplantation Embryos Derived From Immortalized Porcine Cells Via TALE Nucleases
Source: Mol Ther Nucleic Acids. 2014 May 27;3(5):e166–. doi: 10.1038/mtna.2014.15 (PMC4040627; doi:10.1038/mtna.2014.15)
Supplement: Supplementary Figure S5 — Fluorescent PCR results from CMAH KO single cell colonies. [file mtna201415x5.doc]

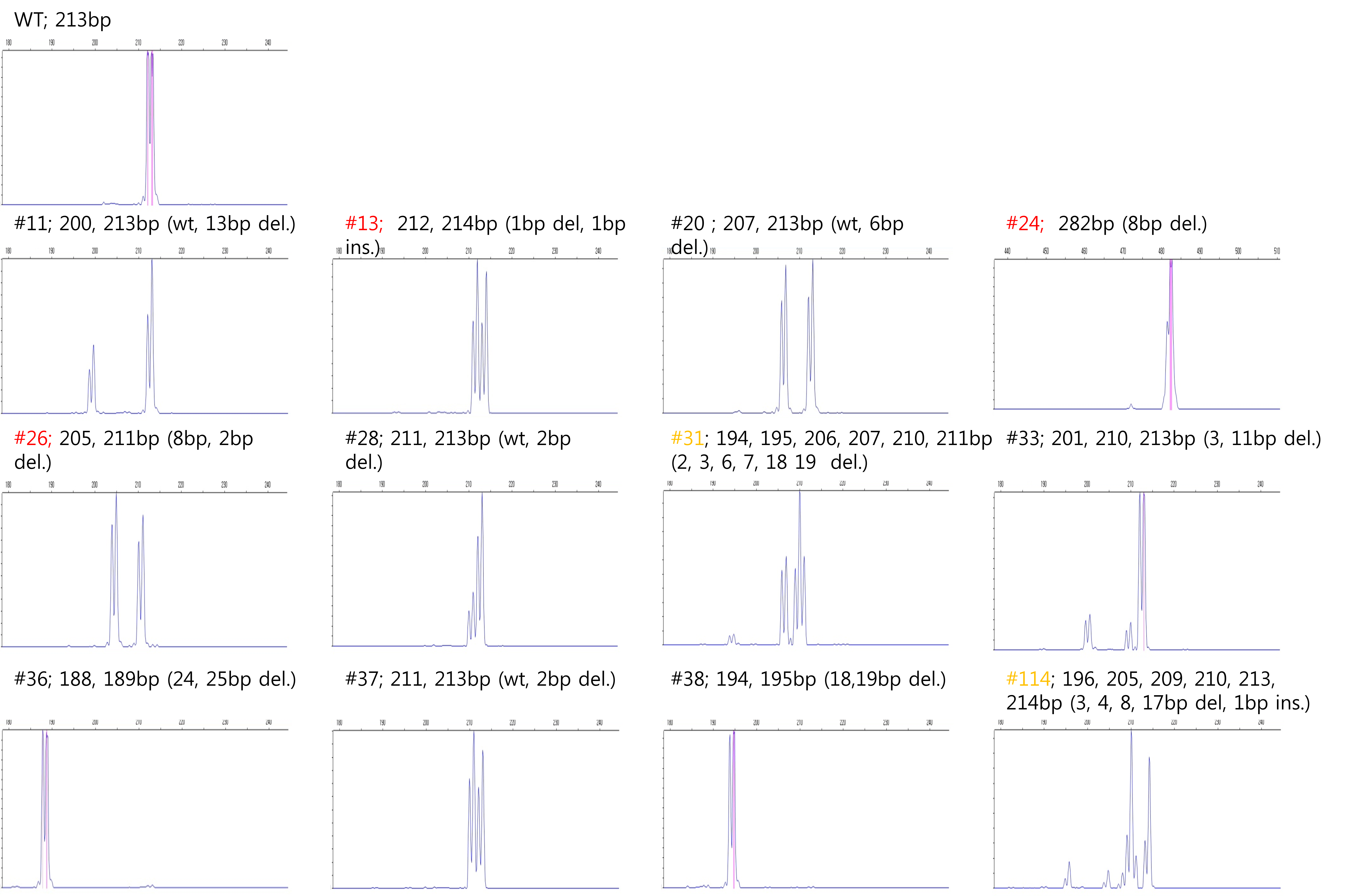


**Figure S5. Fluorescent PCR results from CMAH KO single cell colonies.**

Among them 3 colonies were confirmed that biallelic knock-out colonies those were #13, #24 and #26.
