# Supplementary material for: Production of CMAH Knockout Preimplantation Embryos Derived From Immortalized Porcine Cells Via TALE Nucleases
Source: Mol Ther Nucleic Acids. 2014 May 27;3(5):e166–. doi: 10.1038/mtna.2014.15 (PMC4040627; doi:10.1038/mtna.2014.15)
Supplement: Supplementary Figure S6 — Inserted 282 bp sequences in #24 colony. [file mtna201415x6.doc]

**AGTTCTAGCCACTAGACCACCAGGGAACTCCCTATTCTAAATTCTTGAGCACATTATTTAGGAACCTCAGGAACTTGGCAAGGATTACAAGGAAATATATCTAGATTTAAAAAAAAATCTTTTAACAGAGGTCCCAAAGGAGAGTCATGCACAGCTATGGGAGGAAGTTCAGAAACTGCCCTTGCTACCAGATCACTGTCAGATAAAATGGCCAGCTACATGTTTCTGCACATTGCCCTAAGATCTTTACAAACTTTTCTGTGCATTTTTCCACTTTTAAAA**

**Figure S6. Inserted 282bp sequences in #24 colony.**
