# Supplementary material for: Production of CMAH Knockout Preimplantation Embryos Derived From Immortalized Porcine Cells Via TALE Nucleases
Source: Mol Ther Nucleic Acids. 2014 May 27;3(5):e166–. doi: 10.1038/mtna.2014.15 (PMC4040627; doi:10.1038/mtna.2014.15)
Supplement: Supplementary Figure S7 — SCNT with CMAH KO donor cells. [file mtna201415x7.doc]

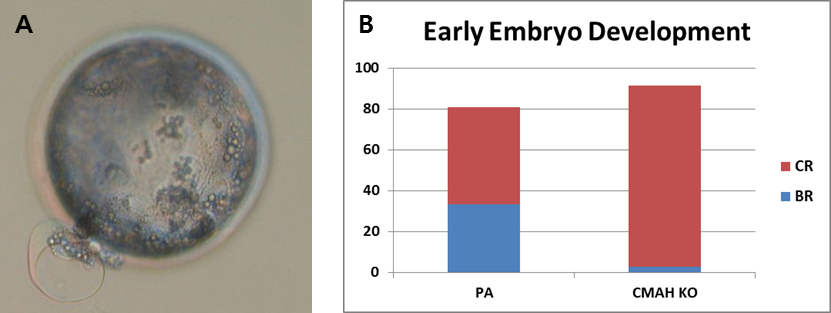


**Figure S7. SCNT with CMAH KO donor cells.**

(A) One blastocyst derived from SCNT with CMAH KO donor cell. (B) Development rates were evaluated in two groups: parthenogenetically-activated embryos (total numbers of oocytes: 69) and SCNT-derived embryos using CMAH KO cells as nuclear donors (total numbers of oocytes: 36). CR: Cleavage Rate, BR: Blastocyst Rate
