# Supplementary material for: Production of CMAH Knockout Preimplantation Embryos Derived From Immortalized Porcine Cells Via TALE Nucleases
Source: Mol Ther Nucleic Acids. 2014 May 27;3(5):e166–. doi: 10.1038/mtna.2014.15 (PMC4040627; doi:10.1038/mtna.2014.15)
Supplement: Supplementary Figures S8 — Illustration of TALEN binding sitesand results of GGTA1-TALEN KO. [file mtna201415x8.doc]

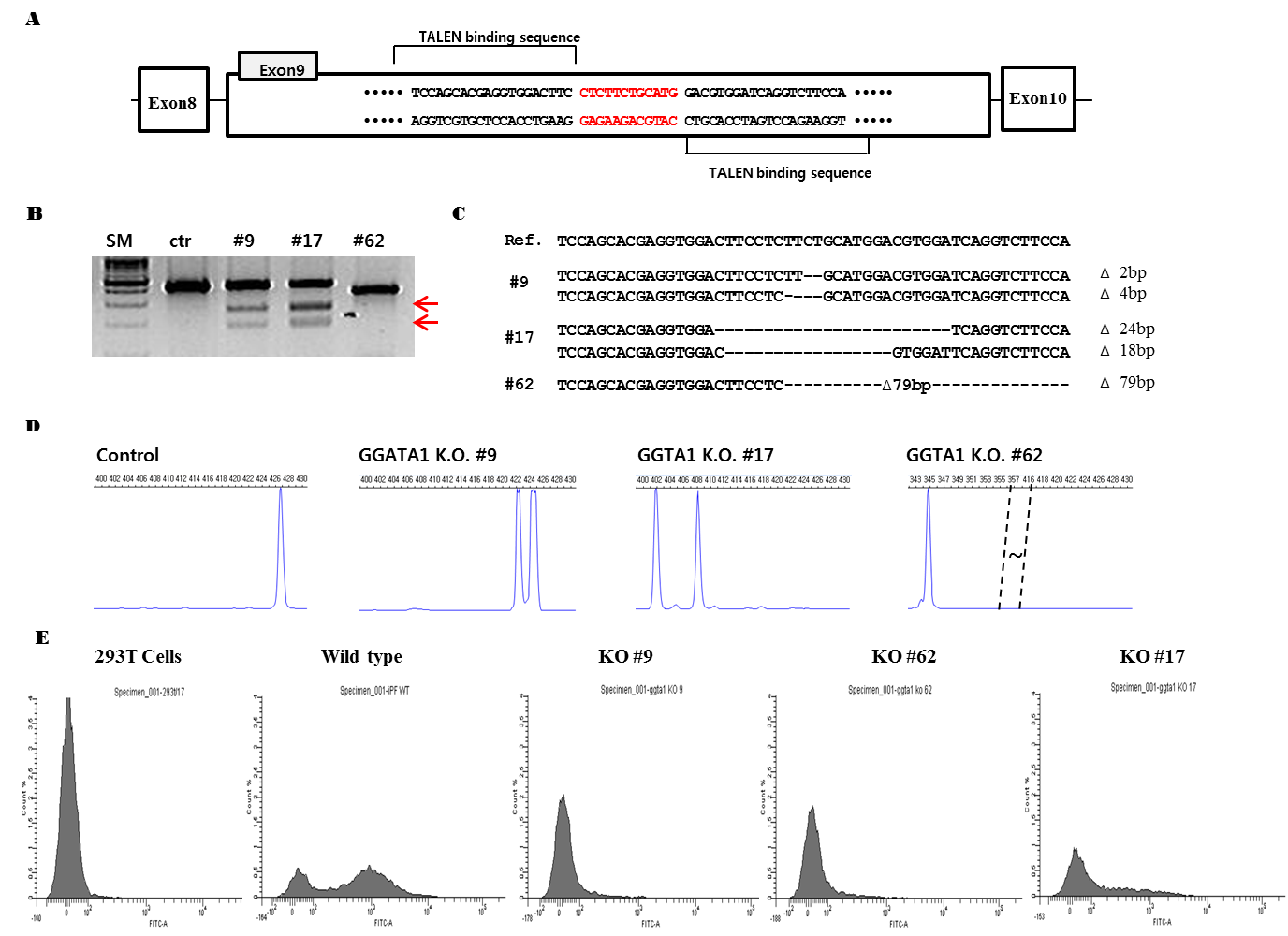


**Figure S8. Illustration of TALEN binding sites and results of GGTA1-TALEN KO.**

(A) DNA-binding sequences and the spacer region for GGTA1-TALEN. (B) T7 endonuclease I (T7E1) assays. (C) DNA sequences of the GGTA1 locus from each GGTA KO clones. (D) Fluorescent PCR (fPCR) assay of the GGTA1 KO clones. (E) FACS analysis of GGTA1 KO clones.
