# Supplementary material for: Production of CMAH Knockout Preimplantation Embryos Derived From Immortalized Porcine Cells Via TALE Nucleases
Source: Mol Ther Nucleic Acids. 2014 May 27;3(5):e166–. doi: 10.1038/mtna.2014.15 (PMC4040627; doi:10.1038/mtna.2014.15)
Supplement: Supplementary Figures S9 — T7E1 assay results from 1st GGTA1 KO single cell colonies. [file mtna201415x9.doc]

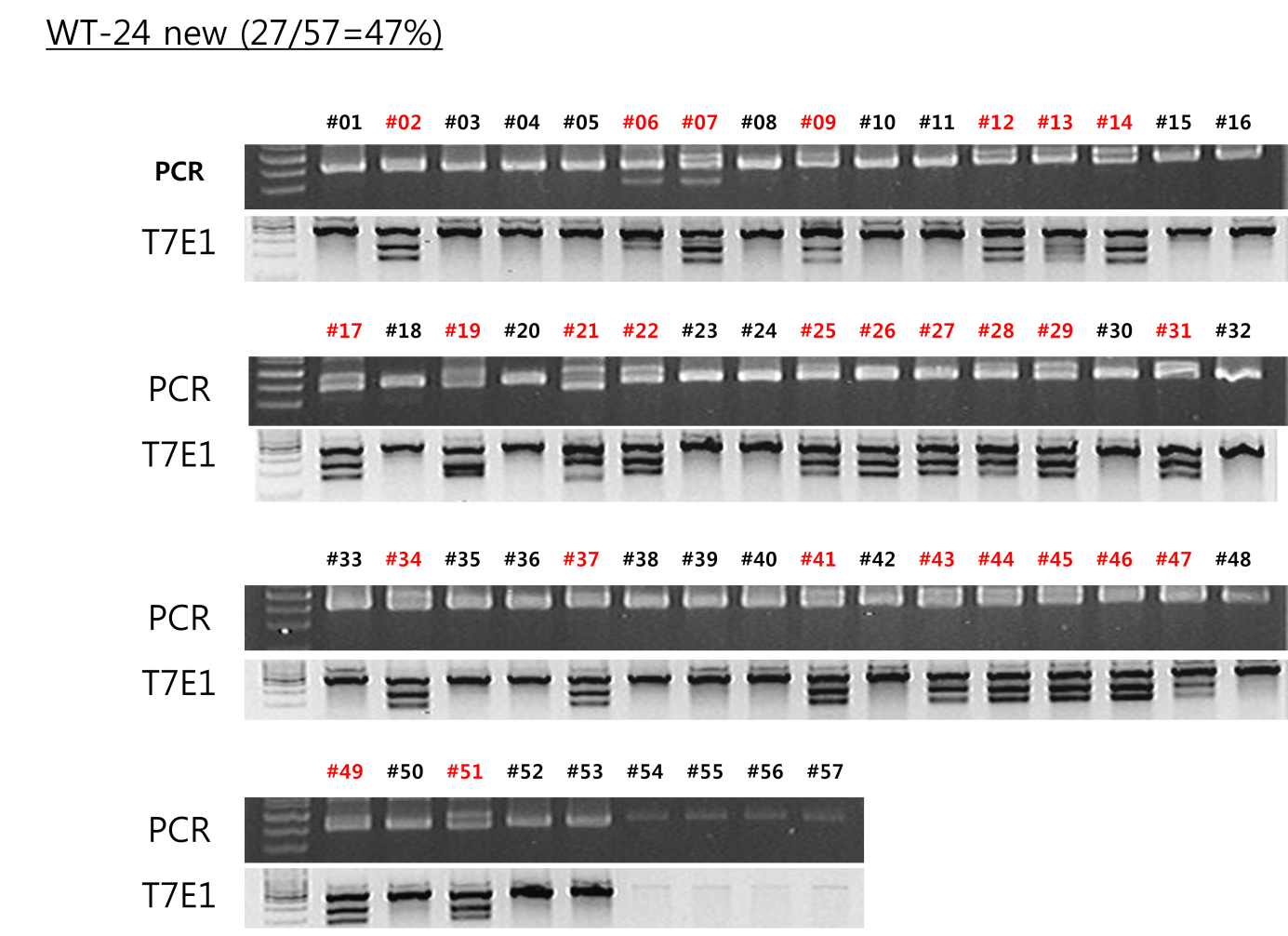


**Figure S9. T7E1 assay results from 1^st^ GGTA1 KO single cell colonies.**

GGTA1 KO single cell colonies were subjected on T7E1 assay for confirming knocked out colonies. Among 57 colonies, 27 colonies were knocked out, which efficiency rate was 47%.
