# Supplementary figures and images for: Production of CMAH Knockout Preimplantation Embryos Derived From Immortalized Porcine Cells Via TALE Nucleases
Source: Mol Ther Nucleic Acids. 2014 May 27;3(5):e166–. doi: 10.1038/mtna.2014.15 (PMC4040627; doi:10.1038/mtna.2014.15)

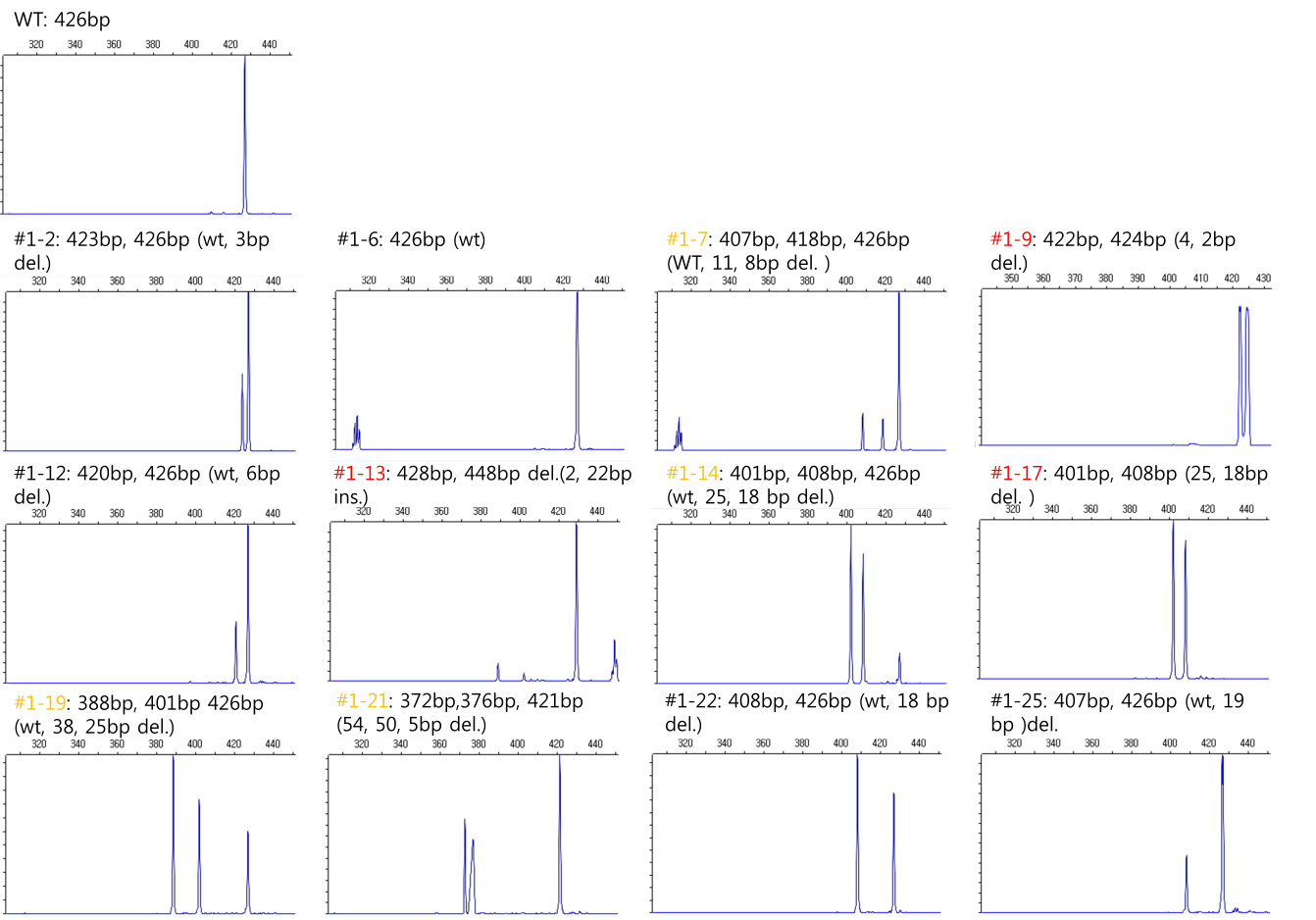


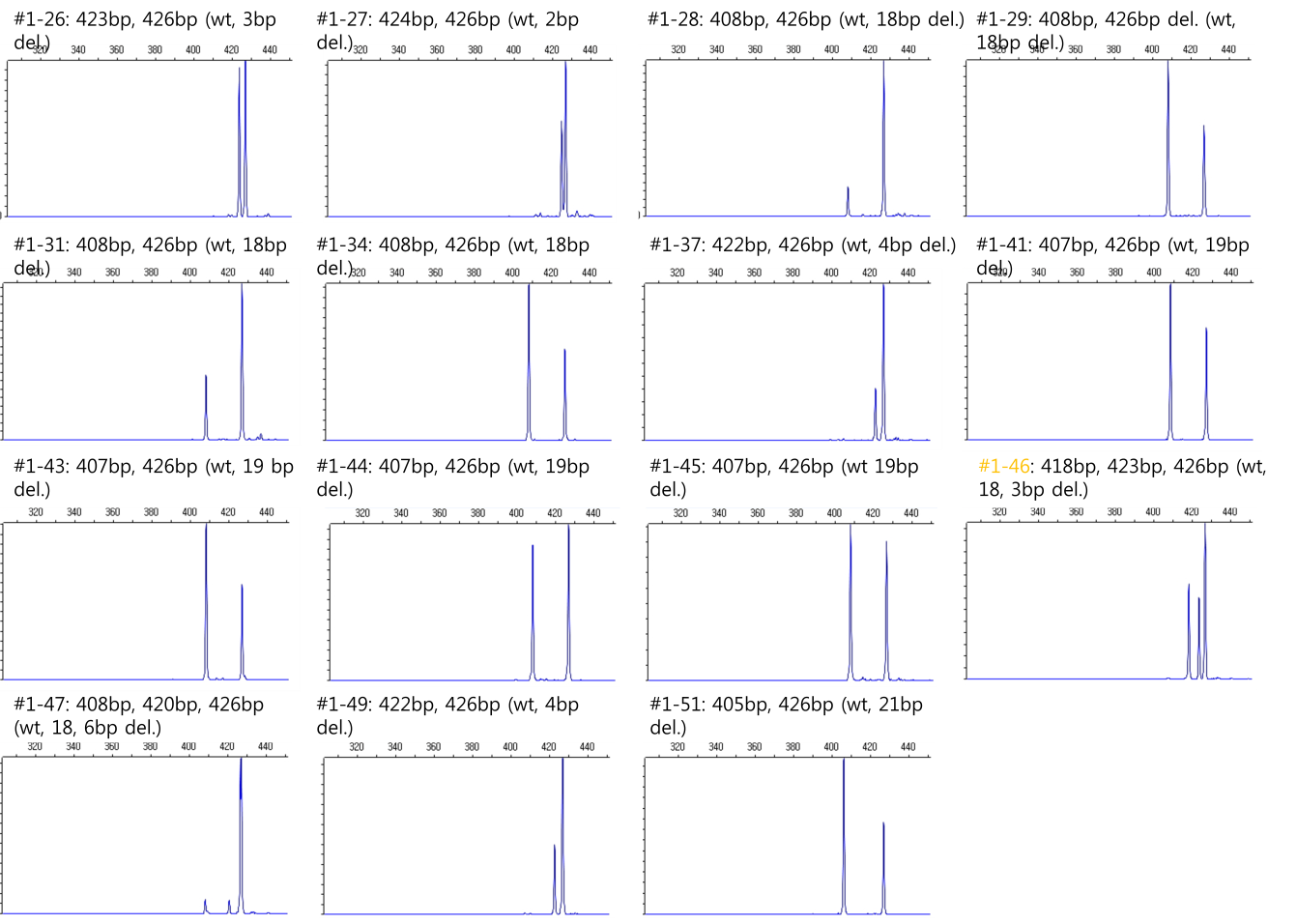


**Figure S10. Fluorescent PCR results from 1st GGTA1 KO single cell colonies.**

Supplement: Supplementary Figures S10 — Fluorescent PCR results from 1st GGTA1 KO single cell colonies. [file mtna201415x10.doc]

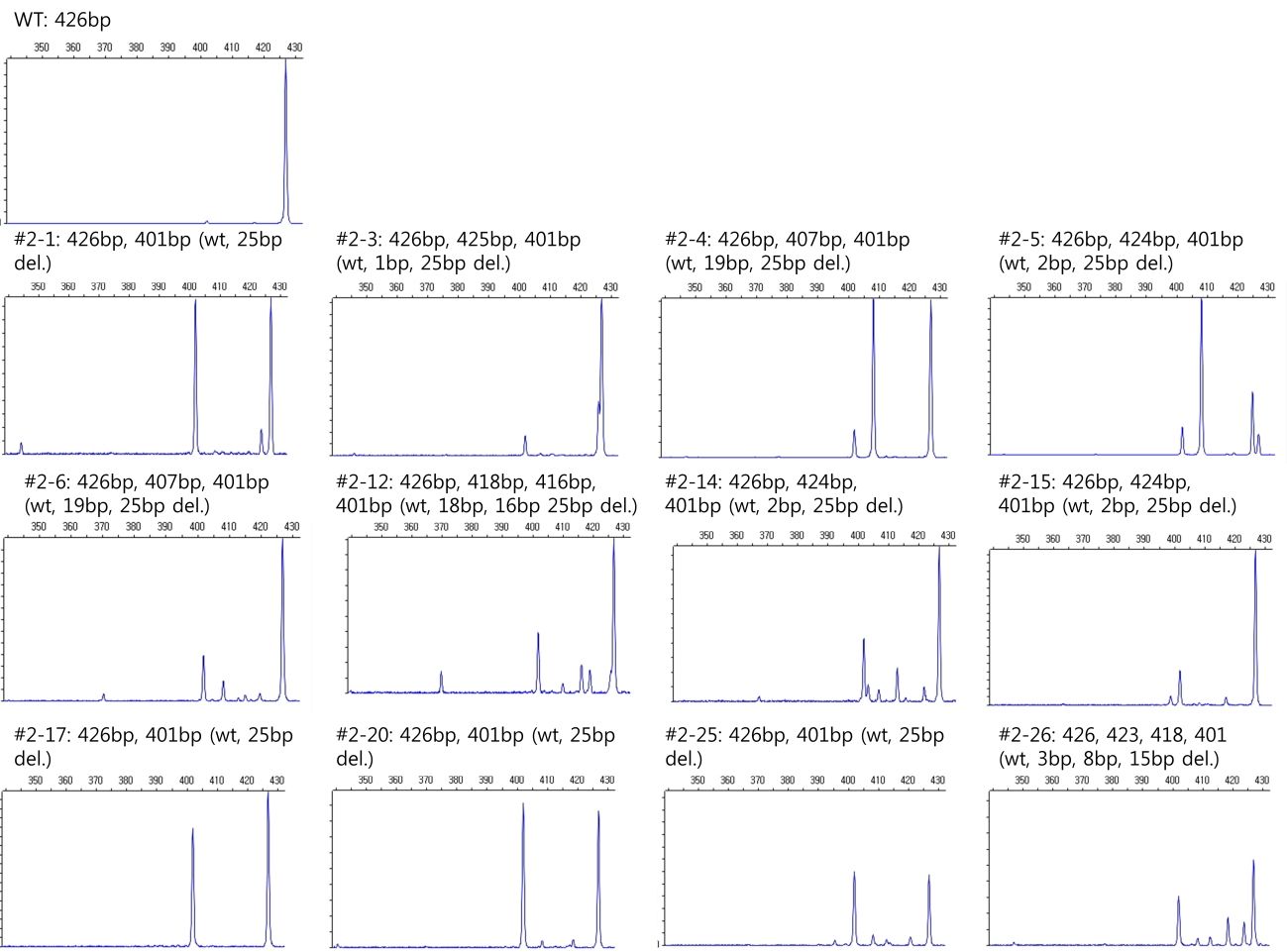


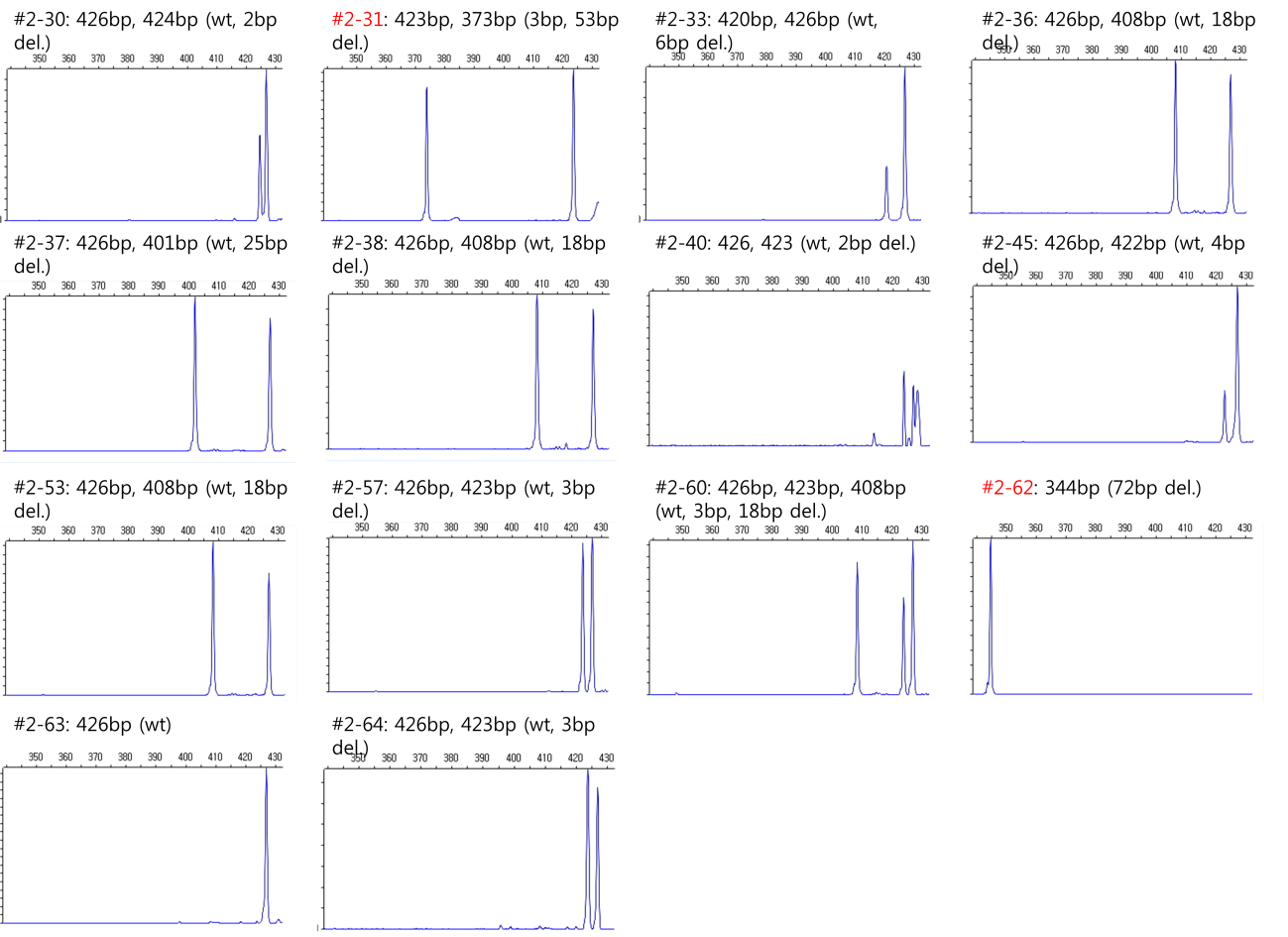


**Figure S12. Fluorescent PCR results from 2nd GGTA1 KO single cell colonies.**

Supplement: Supplementary Figures S12 — Fluorescent PCR results from 2nd GGTA1 KO single cell colonies. [file mtna201415x12.doc]
