# Supplementary material for: Production of CMAH Knockout Preimplantation Embryos Derived From Immortalized Porcine Cells Via TALE Nucleases
Source: Mol Ther Nucleic Acids. 2014 May 27;3(5):e166–. doi: 10.1038/mtna.2014.15 (PMC4040627; doi:10.1038/mtna.2014.15)
Supplement: Supplementary Figures S11 — T7E1 assay results from 2nd GGTA1 KO single cell colonies. [file mtna201415x11.doc]

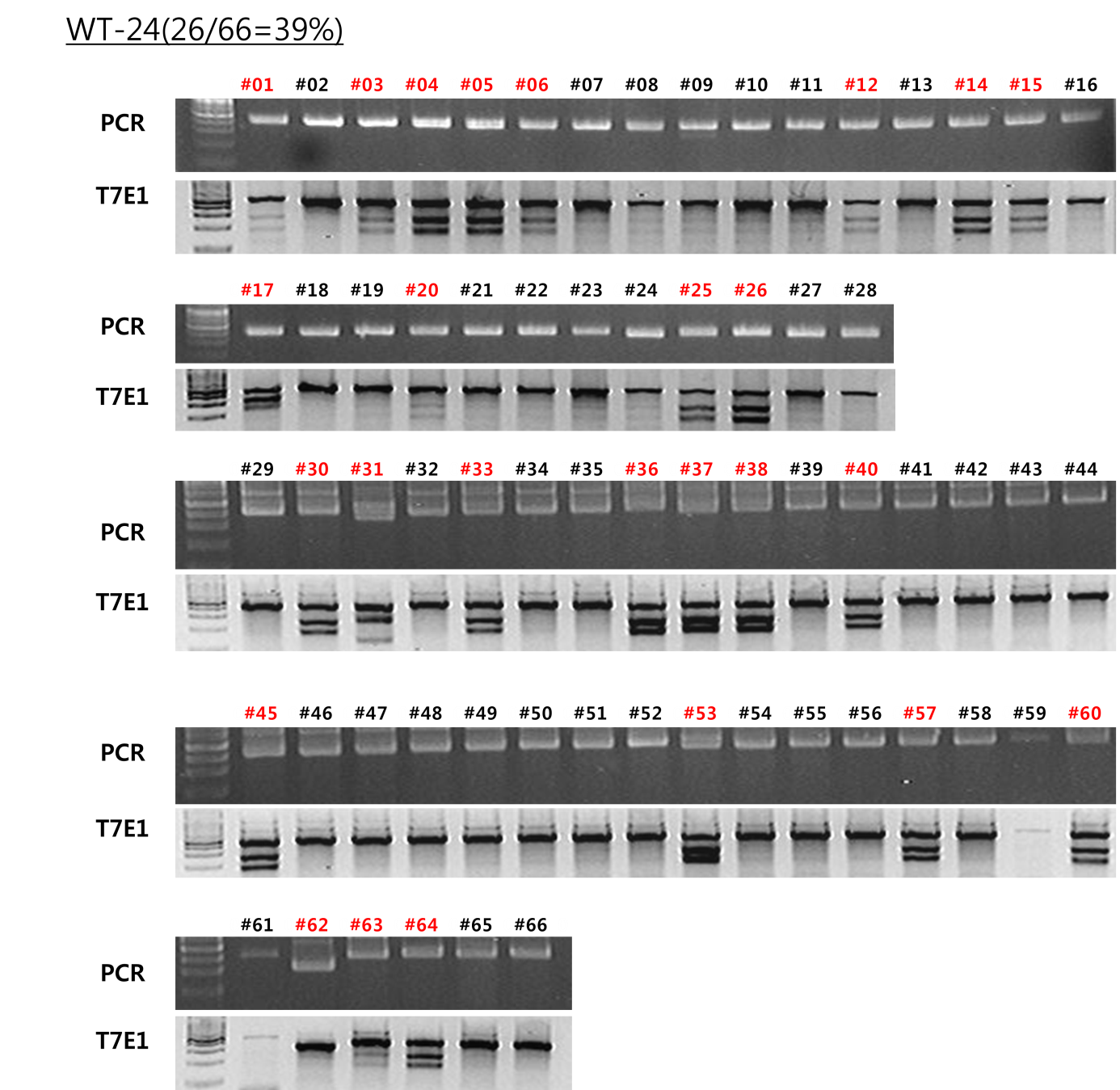


**Figure S11. T7E1 assay results from 2^nd^ GGTA1 KO single cell colonies.**

GGTA1 KO single cell colonies were subjected on T7E1 assay for confirming knocked out colonies. Among 66 colonies, 26 colonies were knocked out, which efficiency rate was 39% .
