# Supplementary material for: Production of CMAH Knockout Preimplantation Embryos Derived From Immortalized Porcine Cells Via TALE Nucleases
Source: Mol Ther Nucleic Acids. 2014 May 27;3(5):e166–. doi: 10.1038/mtna.2014.15 (PMC4040627; doi:10.1038/mtna.2014.15)
Supplement: Supplementary Table S1 — List of primers. [file mtna201415x13.doc]

**Supplementary data**

**Table S1. List of primers.**

| Gene | | Primer sequences (5’-3’) | | Size of PCR product (bp) | GenBank accession no. |
| --- | --- | --- | --- | --- | --- |
|  |  | Forward | Reverse |  |  |
| hTERT | GTGGTGAACTTCCCTGTAGAAGAC | | GAAACAGGCTGTGACACTTCAG | 250 | NC_000005.9 |
| GAPDH | ACCTGCCGTCTGGAGAAACC | | GACCATGAGGTCCACCACCCTG | 252 | AF017079 |
| 1^st^ CMAH | TTGGTCTTCAGCCCTCATCT | | CTGGTAGCAAGGGCAGTTTC | 743 | NM_001113015.1 |
| 2^nd^ CMAH | TTGGTCTTCAGCCCTCATCT | | ATTTAACATTTCCTTACCTGCAC | 307 | NM_001113015.1 |
| fPCR CMAH | TTGAGCCATGCATTTCTGTC | | ATTTAACATTTCCTTACCTGCAC | 213 | NM_001113015.1 |
