# Supplementary material for: Production of CMAH Knockout Preimplantation Embryos Derived From Immortalized Porcine Cells Via TALE Nucleases
Source: Mol Ther Nucleic Acids. 2014 May 27;3(5):e166–. doi: 10.1038/mtna.2014.15 (PMC4040627; doi:10.1038/mtna.2014.15)
Supplement: Supplementary Table S2 — List of real-time PCR primers. [file mtna201415x14.doc]

**Table S2. List of real-time PCR primers.**

| Gene | | Primer sequences (5’-3’) | | Size of PCR product (bp) | GenBank accession no. |
| --- | --- | --- | --- | --- | --- |
|  |  | Forward | Reverse |  |  |
| Bcl-xl | TGGTGGTTGACTTTCTCTCC | | ATTGATGGCACTAGGGGTTT | 134 | AF216205 |
| BAX | GCCGAAATGTTTGCTGACGG | | CGAAGGAAGTCCAGCGTCCA | 146 | AJ606301 |
| p53  p16  DNMT1  DNMT3a  DNMT3b  GLUT1  LDHA | CCTCACCATCATCACACTGG  CTGGACACTTTGGTGGTCCT  TCGAACCAAAACGGCAGTAG  CTGAGAAGCCCAAGGTCAAG  AGTGTGTGAGGAGTCCATTGCTGT  GCTTCCAGTATGTGGAGCAACT  ATCTTGACCTATGTGGCTTGGA | | GGCTTCTTCTTTTGCACTGG  GCGGGATCTTCTCCAGAGTT  CGGTCAGTTTGTGTTGGAGA  CAGCAGATGGTGCAGTAGGA  GCTTCCGCCAATCACCAAGTCAAA  AAGCAATCTCATCGAAGGTCC  TCTTCAGGGAGACACCAGCAA | 213  185  215  238  133  132  214 | NM_213824  AJ316067  DQ060156.1  NM_001097437.1  NM_001162404.1  X17058.1  NM_001172363.1 |
| GAPDH | TCTCTGCTCCCTCCCCGTTC | | TGGCAATGCACGGAACACAC | 51 | AF017079 |
